# The “Dry-Lab” Side of Food Authentication: Benchmark of Bioinformatic Pipelines for the Analysis of Metabarcoding Data

**DOI:** 10.3390/foods13132102

**Published:** 2024-07-01

**Authors:** Gabriele Spatola, Alice Giusti, Andrea Armani

**Affiliations:** Department of Veterinary Sciences, University of Pisa, 56124 Pisa, Italy; alice.giusti@vet.unipi.it (A.G.); andrea.armani@unipi.it (A.A.)

**Keywords:** Next Generation Sequencing Technologies, bioinformatic analysis, foodstuff authentication, DADA2, Geneious Prime, Amplicon Sequence Variants (ASVs), Operational Taxonomic Units (OTUs)

## Abstract

Next Generation Sequencing Technologies (NGS), particularly metabarcoding, are valuable tools for authenticating foodstuffs and detecting eventual fraudulent practices such as species substitution. This technique, mostly used for the analysis of prokaryotes in several environments (including food), is in fact increasingly applied to identify eukaryotes (e.g., fish, mammals, avian, etc.) in multispecies food products. Besides the “wet-lab” procedures (e.g., DNA extraction, PCR, amplicon purification, etc.), the metabarcoding workflow includes a final “dry-lab” phase in which sequencing data are analyzed using a bioinformatic pipeline (BP). BPs play a crucial role in the accuracy, reliability, and interpretability of the metabarcoding results. Choosing the most suitable BP for the analysis of metabarcoding data could be challenging because it might require greater informatics skills than those needed in standard molecular analysis. To date, studies comparing BPs for metabarcoding data analysis in foodstuff authentication are scarce. In this study, we compared the data obtained from two previous studies in which fish burgers and insect-based products were authenticated using a customizable, ASV-based, and command-line interface BP (BP1) by analyzing the same data with a customizable but OTU-based and graphical user interface BP (BP2). The final sample compositions were compared statistically. No significant difference in sample compositions was highlighted by applying BP1 and BP2. However, BP1 was considered as more user-friendly than BP2 with respect to data analysis streamlining, cost of analysis, and computational time consumption. This study can provide useful information for researchers approaching the bioinformatic analysis of metabarcoding data for the first time. In the field of food authentication, an effective and efficient use of BPs could be especially useful in the context of official controls performed by the Competent Authorities and companies’ self-control in order to detect species substitution and counterfeit frauds.

## 1. Introduction

Food authentication is the process which verifies that a food is following its label description [1], and it is essential to tackle fraudulent practices.

Among these practices, intentional species substitution can cause economic losses, ecological impacts, and disrespect of consumers’ religious or ethical beliefs; it can also represent a health risk in the case of illicit presence of toxic or allergenic species [1]. Nowadays, DNA-based methods are widely used in food authentication [1,2,3]. Among these methods, DNA barcoding is the most applied. This method relies on the amplification by polymerase chain reaction (PCR) of specific DNA regions (barcodes) by universal primers. Amplicons are then sequenced by Sanger sequencing (i.e., first generation sequencing), and sequences are compared with a genetic reference database for their taxonomic assignment [4,5,6]. Being based on Sanger sequencing [7], a method with low-throughput capacity, the DNA barcoding efficiency is limited by the number of target species that can be simultaneously detected. For this reason, it is widely recognized as unfit for the authentication of foodstuffs composed of a mixture of species [2]. 

Next Generation Sequencing Technologies (NGS), high-throughput methods able to simultaneously sequence millions of DNA molecules, have opened interest perspectives for the analysis of complex food matrices. Compared with Sanger sequencing, NGS has superior accuracy, sensitivity, and detection efficiency [8]. Metabarcoding, also called amplicon sequencing [9], based on NGS, involves the amplification of barcodes and their sequencing using NGS. The analytical workflow is typically composed of a wet-lab phase (i.e., sample processing steps) and a dry-lab phase (i.e., the data analysis). The first includes DNA extraction, library preparation (amplification of barcodes in a preliminary PCR using dedicated primers), library quantification (usually by fluorometric tools), average size evaluation (usually by capillary electrophoresis), and final library normalization to standard concentration for uploading to the sequencing instrument. Currently, major NGS companies have provided detailed official protocols for this phase. For instance, the “16S Metagenomic Sequencing Library Preparation” protocol is reported among Illumina support guides.

Once libraries are sequenced, the results are returned by the instrument in FASTQ files, intended as strings of A, C, G, T characters and an associated Phred quality score (QS), measuring the nucleotide bases’ calling accuracy. 

The subsequent dry-lab phase refers to the bioinformatic analysis, typically articulated in following steps from data filtering based on the QS, removing artifacts (i.e., chimera), up to the final definition of features by clustering or denoising [10,11,12,13]. Once obtained, features are taxonomically assigned by comparison against reference genetic databases [2,14,15,16]. 

All this is carried out using a bioinformatic pipeline (BP), which is a set of connected algorithms that are executed in a pre-defined order to process and analyze NGS data [17]. BPs therefore play a crucial role in the accuracy reliability and the interpretability of the NGS results. In the context of food authentication, results reliability is especially crucial in the context of official controls performed by the Competent Authorities or where the results are used in judicial proceedings.

Contrary to the wet-lab phase, protocols have not yet been provided for the dry-lab phase, and the major NGS companies provide dedicated cloud-based platforms for data management, storage, and analysis (e.g., BaseSpace Sequence Hub by Illumina) in which the possibility to adjust settings according to the user’s need is limited. On the other hand, a multitude of BPs have been developed.

Factually, learning to use a BP could be time consuming and require important informatic skills, which represents a notable change in operations for many laboratories [18]. Additionally, the wide range of available BPs can make the selection of the most suitable one for a precise need challenging. BPs are usually classified in the literature according to several criteria [14], including the level of customization, the feature typology, and the users’ interface (details in Table 1).

With respect to the feature typology, clustering and denoising represent distinct approaches for grouping reads obtained after the sequencing [10,11,12,13]. *De novo* clustering is a type of hierarchical clustering that employs sequence similarity in order to group sequences into Operational Taxonomic Units (OTUs) without relying on reference databases [12,13]. This approach can be computationally efficient but may overlook subtle sequence variations [10,11,14]. The denoising approach utilizes algorithms to identify unique Amplicon Sequence Variants (ASVs) within a sample [10,11,12]. This method offers higher resolution by distinguishing sequence variants that are potentially grouped into the same OTU using *de novo* clustering [10,11,14]. 

Comparisons between BPs characterized by different feature typologies (OTUs vs. ASVs) in the analysis of metabarcoding data are reported in the literature [19,20,21,22,23,24]. In the field of food inspection, BP comparisons were performed in studies applying metabarcoding to the analysis of microbial communities.

To the best of our knowledge, only three studies compared BPs in the analyzing of data from animal foodstuff authentication [25,26,27], likely due to the fact that the application of metabarcoding to this purpose is still rather limited [2] (Table 2). Denay et al. ([27] and Kappel et al. [25] compared qualitatively and/or quantitatively OTU-based and ASV-based BPs in the analysis of meat samples (Table 2); Klapper et al. [26] compared the results of the sample compositions in canned tuna of six different BPs (OTU-based and ASV-based) (Table 2).

According to Denay et al. [27] and Kappel et al. [25], analyses performed using ASV-based (particularly the DADA2 algorithm) BPs presented advantages with respect to BPs applying OTU clustering.

In two studies previously performed by our research team, metabarcoding was applied to the authentication of seafood products (fish burgers—FBs) [5] and novel foods (insect-based products—IBPs) [28], respectively. In both studies, sequencing data obtained from 16s rRNA metabarcoding on Illumina platforms were analyzed with the open-access DADA2 R package [10] that, according to BP classification, can be considered as an example of a customizable, ASV-based CLI BP. In both these studies species substitution were detected [5,28].

In the present study, we compared the results obtained in the aforementioned studies with those obtained by analyzing the same sequencing data with another BP obtained by the combination of different open-source tools available in the commercial software Geneious Prime v.2024.02 (Dotmatics; Boston, MA, USA) (customizable but OTU-based and GUI). To the best of our knowledge, this BP has never been applied to food authentication. This comparison especially aimed to evaluate differences in the final sample compositions with respect to the BP used. In addition, differences in terms of BP-friendly usability (computational skills and system requirements, data analysis streamlining, cost of analysis, and computational time consumption) were evaluated to provide useful information for researchers approaching the bioinformatic analysis of metabarcoding data for the first time.

## 2. Materials and Methods

### 2.1. Input Data

Sequencing data from 24 FB samples (belonging to nine products) and 45 IBPs samples obtained from two previous studies (study 1 and study 2) [5,28] were used. The FB and IBPs samples were sequenced using Illumina NovaSeq and Miseq instruments, respectively, with a 150-bp paired-end model [5,28]. The sequencing data were represented by folders containing 48 FASTQ files for FB and 90 for IBPs. These FASTQ files were divided into R1 files containing forward reads (24 for FB and 45 for IBPs) and R2 files containing reverse reads (24 for FB and 45 for IBPs). Detailed sequencing data with respect to the total analyzed reads, minimum and maximum reads per sample, and average reads per sample are reported in the results section (Section 3.1.1).

### 2.2. Bioinformatic Pipelines Application for the Analysis of Sequencing Data

#### 2.2.1. First Bioinformatic Pipeline (BP1)

The first BP (customizable, ASV-based, and a command-line interface BP) was the open-access DADA2 R package (BP1) already used in the previous studies [5,28]. This BP was launched again to measure the analysis computational time (see Section 2.4). To highlight that, in this study, the steps performed in BP1 were described in greater detail. Both R1 and R2 were first filtered and trimmed. In particular, in the function -*filterAndTrim()*, the arguments *truncLen*, *minQ*, and *trunQ* were adjusted based on the plot quality profiles. The filtered R1 and R2 were used to train the error model using a machine-learning approach with -*learnErrors()*. Using -*derepFastq()*, R1 and R2 were dereplicated to generate unique sequences. Using -*dada()*, R1 and R2 were denoised (collapsed) in the ASVs by applying the trained error model. Then, they were merged with -*mergePairs()*, and chimera sequences were checked and removed with -*removeBimeraDenovo().* A final ASV table indicating the overall ASV numbers and the relative sequence abundances per sample was produced. Taxonomic assignment was performed using remote BLASTn against the GenBank database (https://blast.ncbi.nlm.nih.gov/Blast.cgi, accessed on 1 April 2024) with a minimum of five hits for each ASV. The minimum percentage identity and the minimum query coverage were set based on those used in the previous studies [5,28]. The BLASTn results were collected in a taxonomy table edited in R version 4.3.2 (R Core Team, 2023). For each sample, the sequence abundance of the identified species (or higher taxonomic rank) in the sample (number of sequences for species/total of sample sequences × 100) was reported.

#### 2.2.2. Second Bioinformatic Pipeline (BP2)

The second BP was a customizable, OTU-based, and graphical user interface BP (BP2). For this analysis, different tools available in the commercial software Geneious Prime v.2024.02 (Dotmatics; Boston, MA, USA) (BP2) were used. The R1 and R2 files were imported together on the software to pair the sequences and create single-paired reads. Subsequently, the tool “*BBDuk*” was used to perform a quality trimming, based on the quality plots obtained in BP1. Then, single-paired reads were merged using the “*BBMerge*” tool, setting a “high merge rate” as reported in the software. A “*de novo assembly*” was carried out using the trimmed and merged filtered reads, prepared by the previous steps, to cluster 97% related sequences into separate contigs (the consensus sequence for each contig represents an OTU).

All the OTUs were taxonomically assigned using the Geneious tool called “*BLAST*” and performing BLASTn against the GenBank database (https://blast.ncbi.nlm.nih.gov/Blast.cgi, accessed on 1 April 2024) using the same identity percentages and query coverages used for BP1. Finally, taxonomy tables containing the sequence abundances of the identified species (or higher taxonomic rank) contained in each sample (number of sequences for species/total of sample sequences × 100) were produced using the Geneious “*Sequence Classifier*” tool and edited in R version 4.3.2 (R Core Team, 2023).

### 2.3. Output Data Analysis

#### 2.3.1. Comparison of Retained Sequences and Number of Features

The overall percentage of retained sequences (range and average) and the overall number of features (ASVs or OTUs) were reported for each BP and compared.

#### 2.3.2. Data Filtering, Sample Composition Comparison, and Statistical Analysis

By setting a sequence threshold based on positive control data, it is possible to detect and discard potential contaminants introduced during the analytical process [2,29]. In the previous studies by Giusti et al. [5,28], sequence abundance thresholds were established using positive control samples: 3.33% for the FB dataset and 1% for the IBPs dataset. Considering that data from positive controls were not used in the present study, and that FB samples revealed no sequences with an abundance between 1% and 3.33%, the threshold was therefore harmonized to 1% for both FB and IBPs data filtering.

After normalization to 100, the composition of each sample was pairwise compared statistically using R version 4.3.2 (R Core Team, 2023). First, the data distribution was evaluated with a Shapiro–Wilk test. Then, the presence of significant differences in sample compositions were assessed using a pairwise Wilcoxon signed-rank test, and a *p*-value < 0.05 was considered indicative of significative differences between evaluated groups.

#### 2.3.3. Alpha Diversity Indices: Shannon Index and Species Richness

Alpha diversity indices, namely the Shannon–Weaver or Shannon–Wiener index (Shannon index) and Species Richness (SR), were computed through “Vegan” version 2.6.4 R package [30]. Then, after the data distribution evaluation with a Shapiro–Wilk test, results were compared using a pairwise Wilcoxon signed-rank test as previously reported.

### 2.4. Bioinformatic Pipelines Friendly Usability Evaluation and Comparison

BP1 and BP2 were evaluated for their “friendly usability”, considering four criteria: computational skills requirement (C1), data analysis streamlining (C2), cost of analysis (C3), and computational time consumption (C4). For each criterion, different sub-criteria (SC) were established, and scores from 0 to 1 were assigned to the BPs (Table 3), with a maximum achievable score of seven. Regarding C4, the computational time employed from the FASTQ files acquisition to the taxonomic assignment was measured and compared. Results were then provided as overall time and divided for time before and after the taxonomic assignment phase. All the analyses were conducted using a PC with 8 cores–processors and 16 GB of RAM running Windows 11 Home.

## 3. Results and Discussion

### 3.1. Output Data Analysis

#### 3.1.1. Retained Sequences and Number of Features

Percentages of retained sequences of 92.0% and 91.2% after applying BP1 and BP2 were observed, respectively, by analyzing data obtained from study 1. These percentages were 93.9% and 81.3%, respectively, in data from study 2 (Table 4). These results are in accordance with those by Kappel et al. [25], who observed that retained sequences were higher after applying an ASV-based BP (DADA2 algorithm) compared with an OTU-based BP.

In terms of the number of features, OTUs had more than ASVs for data from both study 1 and 2 (Table 4). Accordingly, it was observed that the ASV approach (based on denoising) significantly reduces the number of false features [31], which are likely to be artifacts [32,33]. Thus, ASV results in a fewer number of features but closer to the true composition of the sample [27]. Indeed, as observed by the DADA2 developers, ASVs provide a biologically informative and precise resolution—something which is lost during the clustering process that generates OTUs [10,11]. However, even though denoising is effective in removing false features, distinguishing true signals from noise in rare ASVs can be challenging, resulting in the erroneous discharging of rare taxa [14]. This limitation was observed in studies on microbial communities, and it could not be stated for food authentication studies. Indeed, Kappel et al. [25] highlighted that DADA2 revealed fewer false-positive species in foodstuff mock mixture samples with respect to OTU-based approaches, showing that the discharging of rare taxa in that context was not erroneous.

Moreover, environmental samples exhibited higher species richness, resulting in higher values of alpha diversity indices than foodstuff samples. Consequently, erroneous discharge of rare taxa could significantly affect the analysis results. Conversely, the application of a filtering threshold can prevent the desirable detection of rare taxa, such as the reported species involved in mislabeling cases, and thereby avoid their discarding. Thus, the application of filtering threshold could prevent denoising from significantly affecting the accuracy of foodstuff authentication, regardless of their complexity. Denay et al. [27], by comparing the splitting level associated with the *de novo* clustering method (OTU-based approach) and denoising (ASVs approach), observed that the number of features in which each “real” sequence was split was higher when applying the *de novo* clustering method. The splitting level of amplicon sequences is expressed as the log10-fold change between the expected number of taxa in each sample and the number of predicted features. When noise is removed from sequences (denoising), each real sequence is divided into approximately 10 features, indicating moderate splitting [27]. However, when sequences are grouped into new features (*de novo* clustering), the splitting level increases [27]. In summary, the splitting level rises significantly from denoising to de novo clustering, demonstrating the increasing fragmentation of real sequences into features with each method. Therefore, in addition to the aforementioned factors, this may also help to explain why the number of OTUs was greater than the number of ASVs.

#### 3.1.2. Sample Composition and Statistical Analysis

**Figure 1:**
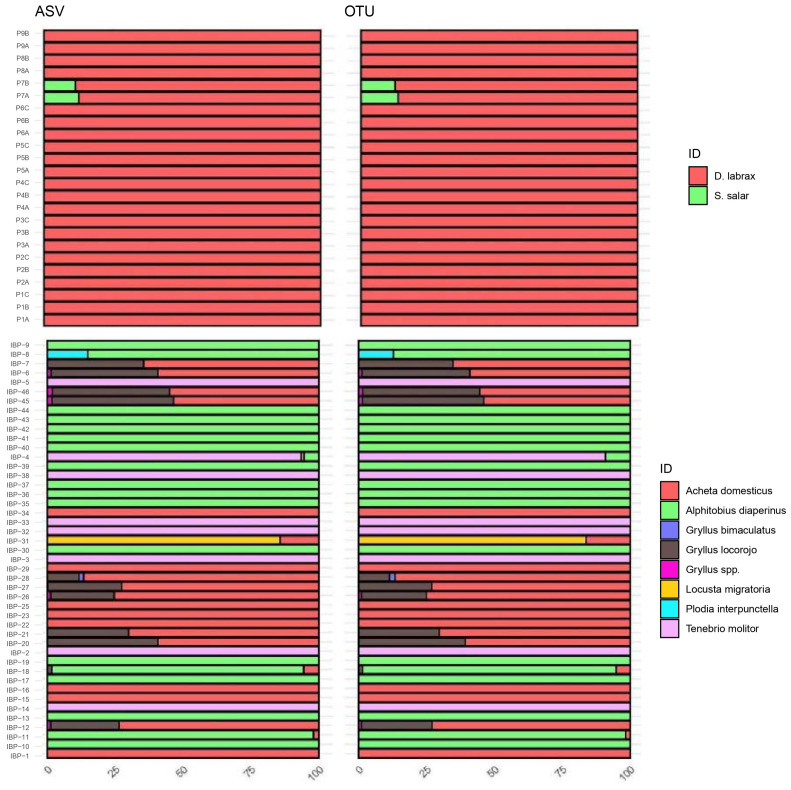
Sample compositions after the application of BP1 (ASV) and BP2 (OTU) to FB and IBP datasets. Each sample composition was represented by a stacked bar plot produced with the sequence abundances of each identified taxa.

The composition of FB samples was almost identical regardless of the BP used. Indeed, only in the 8.3% (2/24) of the FB samples, limited differences occurred in sequence abundances. In detail, the composition of the P7A and P7B samples differed in terms of the abundance of sequences for *Dicentrarchus labrax* and *Salmo salar*, which varied by 0.9% and 1.2%, respectively, after the application of BP1 and BP2 (Figure 1). The composition of each sample (FB and IBPs), in terms of sequence abundances for each assigned species (or higher taxonomic rank) after applying BP1 and BP2, resulted in a non-normal distribution according to the Shapiro–Wilk test (*p*-value < 0.05). Therefore, the Wilcoxon signed-rank test was considered appropriate for comparing the sample compositions, after removing zero values. No significative differences were found comparing the sample compositions with the Wilcoxon signed-rank test (*p*-value = 1.0).

In the case of FB samples, limited differences in sequence abundances of some taxa were observed in 33.3% (15/45) of the cases, with a variation ranging between 0.01% and 3.64% among the different taxa (Figure 1, Table S1) and a mean of 0.76%. However, also in this case, no significative differences were found comparing sample compositions with the Wilcoxon signed-rank test (*p*-value = 0.966). In IBP-4, 1.08% of sequences were associated to *Locusta migratoria* only after the application of BP1 (Figure 1, Table S1). Contrariwise, by applying BP2, the sequence abundance of *L. migratoria* was found to be below the 1% threshold (0.84%), leading to its exclusion during filtering. By applying the same threshold, BP1 was proven to be the best performing with respect to the representation of sample compositions, as also highlighted by other studies on this topic [25,27]. Therefore, a BP2 lower accuracy can be assumed, also considering that *L. migratoria* had a sequence abundance of 0.84%, thereby not highly under the threshold.

Therefore, although no significant differences were observed, the compositions obtained after the application of BP1 could be considered more reliable. In this respect, there has been an increasing development factually of BPs based on the ASV approach, and Callahan et al. [11] even suggest that they should replace OTU-based BPs. Indeed, currently in foodstuff authentication studies, the use of DADA2 has increased in recent years [2].

Several studies applying metabarcoding to the determination of microbial communities compared OTU-based and ASV-based BPs [19,21,24]. While Chiarello et al. [23] found differences in microbial communities analyzed with the OTU vs. the ASV approach, other studies did not highlight significative differences in sample compositions based on the approach [19,21,24]. Overall advantages and disadvantages have been highlighted by using both approaches [11,14,16]. In regards to studies analyzing the species compositions of animal foodstuffs, no significative differences related to the usage of ASV-based BPs or OTU-based BPs were factually highlighted [25,27].

#### 3.1.3. Alpha Diversity Indices: Shannon Index and Species Richness

For samples from study 1, the Shannon index ranged from 0 to 0.38 for the data analyzed with BP1 and from 0 to 0.4 for the data analyzed with BP2. As expected, for both BP1 and BP2, the higher values were found in P7A and P7B, which were duplicates of the same product containing two fish species (*D. labrax* and *S. salar*) [5]. All the other samples showed a Shannon index equal to 0 (because they were found to be composed only of *D. labrax)* while all the other species were assumed to be contaminants and were discarded after the data filtering. The minimal differences observed between the results obtained in this study and those obtained in our previous work [5] could be related to the normalization to 100 performed in this case. However, the Wilcoxon signed-rank test showed no significant differences (*p*-value = 0.98), regardless of whether BP1 or BP2 was used.

**Figure 2:**
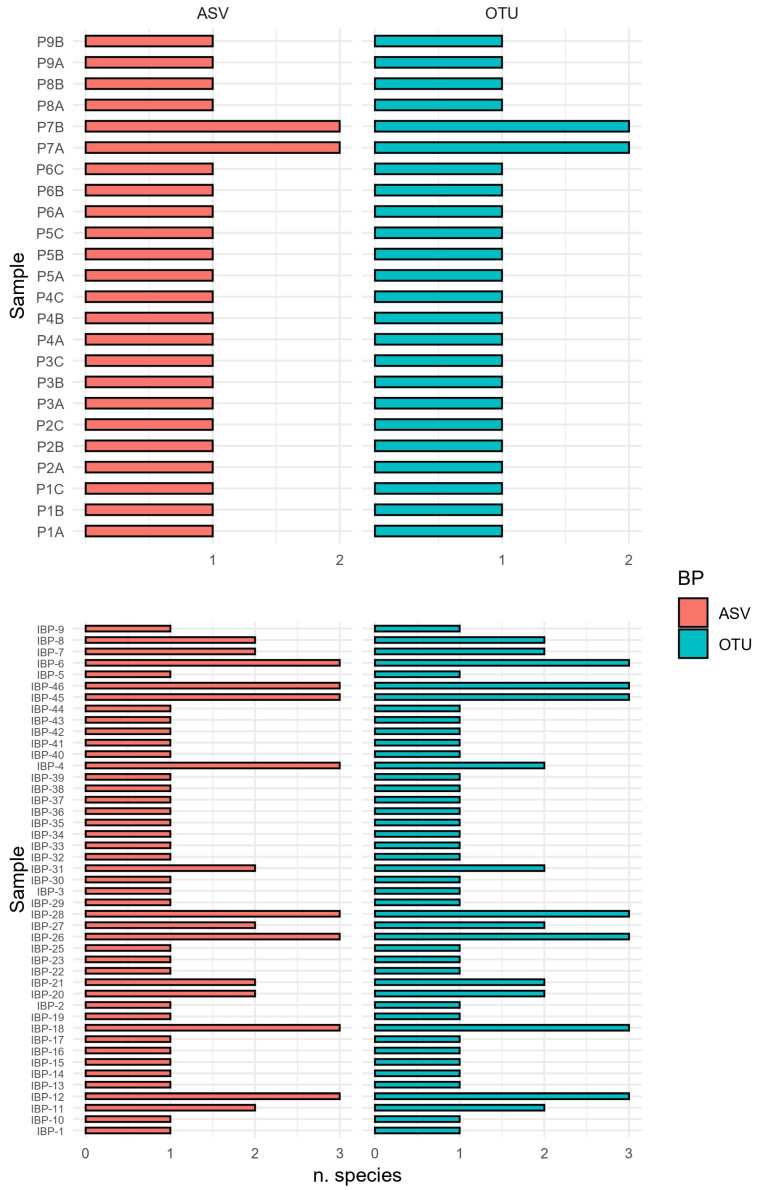
Species richness (number) of FB and IBPs samples analyzed with BP1 (ASV-based) and BP2 (OTU-based).

Regarding species richness, no differences were observed between the data analyzed with BP1 and BP2 in the sample from study 1. This is consistent with the previously reported results for the sample compositions comparison (Section 3.1.2), in which the number of species identified was found to be the same in all samples regardless of the BP used (Figure 2)

For the samples from study 2, the Shannon index of the samples ranged from 0 to 0.76 for the data analyzed with BP1 and from 0 to 0.75 for the data analyzed with BP2. In fact, the Wilcoxon signed-rank test showed no significant differences (*p*-value = 0.93), regardless of the BP used. Regarding species richness, differences were observed only regarding the presence of *L. migratoria* that was observed in IBP-4 only after the analysis performed with BP1 (see Section 3.1.2). More detailed results of species richness were reported in Figure 2.

### 3.2. Bioinformatic Pipelines Friendly Usability Evaluation and Comparison

**Figure 3:**
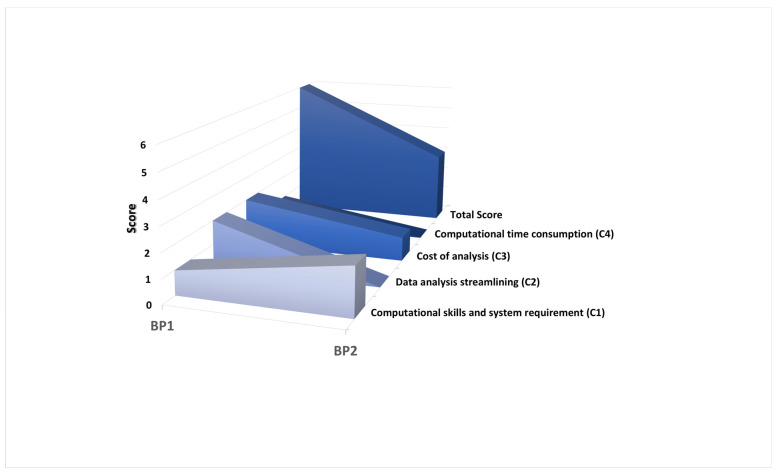
Distribution of the friendly usability score for BP1 and BP2 among the pre-established criteria (C1–C4).

Although the maximum achievable score was not attained, BP1 scored higher than BP2, with six and three (out of the total seven), respectively (Figure 3 and Table 5).

#### 3.2.1. Computational Skills and System Requirements (C1)

BP2 resulted in being more suitable for beginners than BP1 (Table 5). This result is due to the score assigned to SC1a: the fact that DADA2 was developed on R, which works as a CLI, can be intimidating for users with less coding language knowledge. Indeed, installing and using CLI BPs could represent a challenge for non-computer specialists [14,34]. Contrariwise, GUI BPs, without requiring knowledge of programming, make laboratories work easier [18,34]. In this respect, BP2, being structured only with tools available on Geneious Prime (v.2024.02), guarantees to the user a GUI in which the tools appear as visually intuitive environments arrangeable in a clear and accessible manner. Moreover, the GUI of Geneious Prime (v.2024.02) allows to directly visualize raw reads during all BP workflows, reads clustered in each OTU, consensus sequences, and the taxonomy tables produced. On the contrary, the CLI of DADA2 in R only allow to only visualize the number of reads filtered, merged, and denoised. Considering that the application of metabarcoding in foodstuff authentication is still limited, the use of a BP built on Geneious Prime could represent an easier approach for novel users. However, it is interesting to note that, to the best of our knowledge, Geneious Prime has never been used in the context of animal foodstuff authentication but only for studies on environmental DNA [35,36,37,38].

Reviewed studies regarding foodstuff authentication reveal that most researchers in this field used CLI BPs [2]. Nevertheless, the R programming language has become a crucial computational tool for research in fields such as biology, statistics, and medicine [39]. It is often considered an ideal first programming language due to its easy learning curve for beginners [39]. Therefore, it is even more important for those interested in using a bioinformatics tool such as DADA2 [16] to be familiar with it. Indeed, in the field of bioinformatics, Python and R have emerged as the dominant programming languages, and R, in particular, has a strong and potentially long-lasting position within bioinformatics [39]. Nowadays, several solutions guaranteeing a more user-friendly usage of the R programming language are available. For instance, since the absence of a graphical interface is likely the main problem in using R, several different integrated development environments (IDE) specifically designed for data science were developed [39]. An IDE is a software that brings together several useful tools to make programming more efficient. Even though it does not necessarily provide a GUI, it does allow for the combination of both the code and the generated graphical output in the same window. RStudio is a popular open-source IDE currently maintained and promoted by Posit (formerly RStudio Inc., Boston, MA, USA, https://posit.co/, accessed on 15 April 2024). It provides an editor for writing and executing R code available in open-source and commercial editions and runs on the desktop of different operating system (Windows, Mac, and Linux). It includes a console, syntax-highlighting editor that supports direct code execution, and tools for plotting, history, debugging, and workspace management. Another tool developed to facilitate research activity is RMarkdown, an R package that helps researchers create reports and documents that combine text and code. This allows researchers to easily show their results and make their work reproducible. Moreover, as RStudio, another IDE widely used for both Python and R is Jupiter [39]. Finally, the Galaxy Project (https://galaxyproject.org, accessed on 15 April 2024) [40,41,42] offers a user-friendly web interface, even for scientists without programming expertise, due to its user-friendly web GUI which streamlines even large-scale data analysis [42]. It also allows data upload, tool selection, parameter definition, and analysis execution, along with workflow creation via a drag-and-drop interface, making advanced analyses accessible to a broader scientific audience [42]. In addition, the Galaxy Project provides a range of open-source tools for generating BPs. These tools include the functions of the DADA2 R package, which can be used to replicate the DADA2 pipeline (BP1) as it is used in R or RStudio.

#### 3.2.2. Data Analysis Streamlining (C2)

BP2 resulted in being less streamlined than BP1 (Table 5). In fact, BP2 cannot be easily applied to all samples simultaneously (see SC2a). For each sample, a dedicated folder should be created to contain distinct taxonomy tables that show the sequence abundances of all the identified species in that sample. Moreover, the taxonomic assignment using the Geneious Prime (v.2024.02) could be interrupted if a great number of OTUs were blasted simultaneously. Furthermore, Geneious Prime (v.2024.02) does not allow for result plotting or diversity indices calculation (see SC2b). Therefore, the taxonomy table of each sample must be exported and combined with R or RStudio for further analysis.

Conversely, the DADA2 approaches allowed for the processing of large amounts of data simultaneously (SC2a) and for directly analyzing the taxonomy tables outputted in R. In addition, it allowed for the calculation of diversity indices using packages such as phyloseq [43], vegan [30], and tidyverse (tidyr, dplyr, and ggplot2) [44] (SC2b).

#### 3.2.3. Cost of Analysis (C3)

BP2 resulted in being more expensive than BP1 (Table 5). Indeed, the BP2 was built using Geneious Prime (v.2024.02), which is a commercial software program having costs of between $200 and $22,500 per year, depending on the number of users and the typology of buyers (i.e., student, academic, corporate) (https://www.geneious.com/pricing/, accessed on 20 April 2024) (see SC3a). On the contrary, R version 4.3.2 (R Core Team, 2023) is a free software program, widely used for data analysis in several scientific fields [39], that runs on various operating systems, including Linux, Windows, and MacOS. Additionally, R could be considered more versatile as it has no limitations in terms of the number of users. On the contrary, Geneious Prime runs only on main operating systems, such as Linux, Windows, and MacOS. However, it can be used by a maximum of 10 devices. Indeed, many researchers, particularly those in small labs, usually bring their own devices and can significantly benefit from utilizing existing open-source software [34]. With respect to SC3b, free tutorials for the analysis of metabarcoding data are available online for both DADA2 (https://benjjneb.github.io/dada2/tutorial.html, accessed on 25 April 2024) and Geneious Prime (https://www.geneious.com/tutorials/metagenomic-analysis/, accessed on 20 April 2024). In this way, eventual costs needed for specific training are avoided. However, these tutorials are developed for sequencing data of microbial communities Therefore, some adaptations may be necessary depending on the user’s needs, metabarcoding gene target, and study aims.

#### 3.2.4. Computational Time Consumption (C4)

**Figure 4:**
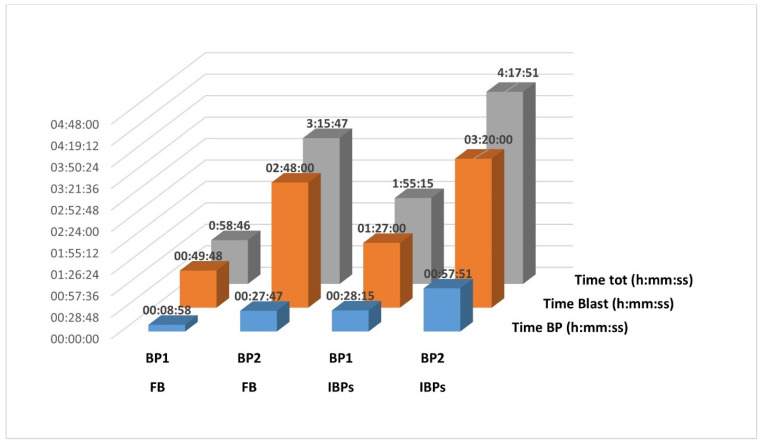
Time consumption for BP1 and BP2. Time of each phase is reported as hours: minutes: seconds. Time BP: is the time needed to complete the BP phases until the taxonomic assignment phase (BLAST). Time Blast: is the time needed to complete the taxonomic assignment phase consisting of a remote BLASTn against the NT GenBank database (https://blast.ncbi.nlm.nih.gov/Blast.cgi, accessed on 1 April 2024). Time tot: is the total time consumption to perform the computational analysis.

BP1 was proven to be faster than BP2. In fact, according to Kappel et al. (2023) [25], our results showed that for both the analysis of samples from study 1 and 2, the overall computational time for BP2 was 3.3 and 2.2 times longer, respectively, than BP1 (Figure 4). The work of Denay et al. [27] confirms this observation, suggesting that this could be due to the splitting level as explained before in Section 3.1.1.

The computational time also strictly depends on the machine used to perform the analysis, causing results from different studies to be poorly compared with each other. In this respect, according to Brandies and Hogg [45], a PC with 32 cores–processors and 128 GB of RAM is usually sufficient to run the most common BPs in a reasonable time. However, these types of hardware requirements are quite expensive. Moreover, even the analysis of a small amount of metabarcoding sequencing data (<50 pairs of R1 and R2 FASTQ files) with DADA2 could be unreliable with basic laptops (e.g., dual-core and ≤8 Gb RAM), requiring High-Performance Computing (HPC) [46]. However, the PC (Section 2.4) used in this study can be considered as sufficiently performant due to the limited size of the data analyzed.

Despite this, it should be noted that, for the analysis of larger datasets (>50–100 pairs of R1 and R2 FASTQ files) in DADA2, the use of HPC facilities equipped with multithread processors and high RAM (≥64 Gb) [46] could be required.

The usage of HPC could also reduce the time consumption linked to the taxonomic assignment phase, which was found as the most time-consuming phase in both BP1 and BP2 (Figure 4). Regardless of all the aforementioned aspects, the speed of the Internet connection and the functionality of NCBI’s servers are the greatest influencing factors. Thus, for clear results and good procession timing, applying BP2 simultaneously for all samples is not recommended. On the contrary, DADA2 could perform the taxonomic assignment against a reference dataset [10] limiting the time needed to perform a remote BLASTn against the NT database of GenBank. In this study, no customized reference datasets were produced or used. In this respect, curated and open-access databases of 16S rRNA sequences, such as SILVA [47,48] and RDP [49,50], exist [51] and are routinely used for the taxonomic assignment of microorganisms. Studies on food authentication must rely on public non-curated databases (e.g., GenBank) or on non-public internal databases.

## 4. Conclusions

Choosing the most suitable BPs in the metabarcoding analysis for foodstuff authentication is of utmost importance. However, the literature focused on this analytical phase is scarce.

In this study, two BPs—used for the analysis of metabarcoding sequencing data from samples of foodstuff of animal origin to evaluate differences in their compositions—were compared. The friendly usability of these BPs was also evaluated and compared, for the first time. Regardless of the similar results in terms of sample compositions, BP1 (customizable, ASV-based, CLI) was found to be better in terms of friendly usability (data analysis streamlining, cost of analysis, and computational time consumption). In this respect, BP1 could be recommended for analyzing metabarcoding data from foodstuff samples of animal origin, offering a good balance between accuracy, speed, and cost-effectiveness. The effective and efficient use of BPs translates in fact to a conscious interpretation of the metabarcoding results, thus contributing to better detection of species substitution phenomena and counterfeit frauds. Overall, this study could provide useful information for researchers approaching the bioinformatic analysis of metabarcoding data for the first time.

This study can be also regarded as a preliminary approach towards the optimization (and standardization) of bioinformatic analysis in metabarcoding applied to foodstuff authentication. Indeed, standardization can allow this technique to enter more and more into the routine controls performed by official bodies and the self-control procedures implemented by food companies.

## Figures and Tables

**Table 1:** BPs classification criteria with detailed descriptions.

Classification Criterion	BP Category	Description
Level of customization	Customizable	Chain of software, tools, or algorithms with commands and settings that can be modified to be adaptable to different users’ needs [14].
	Precompiled	Chain of software, tools, or algorithms with pre-defined and validated commands and settings that usually facilitate the analysis for users with few bioinformatics skills [14].
Feature typology	Operational Taxonomic Units (OTUs)	Present a hierarchical clustering phase in which raw sequences are grouped into OTUs according to their pairwise similarity (*de novo* clustering) [12,13].
	Amplicon Sequence Variants (ASVs)	Present a denoising phase, instead of a clustering phase, in which an error correction algorithm is applied to sequences to produce features [10]. This procedure produces ASVs, which are identical denoised reads with as little as a 1base pair difference between variants [11].
Users’ interface	Command-line interface (CLI/CL)	BP built using software in which commands are typed into a terminal [14].
	Graphical user interface (GUI)	BP built using software in which users interact with graphical icons [14].

**Table 2:** Details of studies comparing the application of different BPs (ASV-based vs. OTU-based) to animal origin foodstuff authentication.

Authors	Tool/Algorithms	Feature Typology	Level of Customization	Users’ Interface	Type of Comparison
Denay et al. [27]	VSearch	OTU-based (95% *de novo* clustering)	customizable	CLI	Workflow performances ^[a]^
	VSearch	OTU-based (97% *de novo* clustering)	customizable	CLI	
	VSearch	OTU-based (100% *de novo* clustering—dereplication)	customizable	CLI	
	DADA2	ASV-based (denoising)	customizable	CLI	
Kappel et al. [25]	VSearch	OTU-based (97% *de novo* clustering)	customizable	CLI	retained sequences (minimum, maximum, mean, DS); features (OTUs, ASVs) number and percentage, sample compositions
	VSearch	OTU-based (100% *de novo* clustering—dereplication)	customizable	CLI	
	DADA2	ASV-based (denoising)	customizable	CLI	
Klapper et al. [26]	QIIME (DADA2)	ASV-based (denoising)	customizable	CLI	sample composition
	Galaxy (DADA2)	ASV-based (denoising)	customizable	GUI	
	Galaxy (VSearch)	OTU-based (97% *de novo* clustering)	customizable	GUI	

^[a]^: i.e., Observed and expected compositions of the samples at the genus level; average precision calculation; yield of the analysis as the number of retained reads through taxonomic assignment; and calculation of the Euclidean distance error reflecting how far predictions are from the expected compositions of the samples. [27]. *Tool*/*Algorithms*: This column reports the tools and/or algorithms mainly used to generate the BP used to analyze the sequencing data. *Feature typology*: Report whether the BP was ASV-based or OTU-based. For OTU-based BP, percentage similarity identity was also reported.

**Table 3:** Established sub-criteria to score the friendly usability of BP1 and BP2.

Criteria	Sub-Criteria (SC)	Score 0	Score 1
Computational skills and system requirement (C1)	Is the pipeline available on Windows? (SC1a)	No	Yes
	Do you need to have any programming experience to use the pipeline? (SC1b)	Yes	No
Data analysis streamlining (C2)	Can the BP be easily applied to all samples simultaneously? (SC2a)	No	Yes
	Is it possible to perform output data analysis (i.e., diversity index and plotting of results) on the software hosting the BP? (SC2b)	No	Yes
Cost of analysis (C3)	Is the software used for hosting BPs free of charge? (SC3a)	No	Yes
	Are there any free tutorials available for using the pipeline? (SC3b)	No	Yes
Computational time consumption (C4)	Which is the faster BP? (SC4a)	Slower	Faster

**Table 4:** Sequencing data details. *Total analyzed reads* refers to the sum of reads obtained during the sequencing for each sample in the sequencing dataset. *Retained reads refers* to the percentage of reads resulting after the application of BP on the sequencing data. *N. features* correspond to the number of features (ASVs or OTUs) produced with the application of BP1 or BP2.

Sequencing Datasets	BP	Total Analyzed Reads	Min–Max Reads for Sample	Average Reads for Sample	Min–Max Retained Sequences (%)	Average Retained Reads (%)	N. Features
FBs	BP1 (ASVs)	2,264,053	25,006–247,583	94,336	73.8–96.8	92.0	65
FBs	BP2 (OTUs)				76.6–97.5	91.2	287
IBPs	BP1 (ASVs)	1,461,601	2312–123,871	32,408,02	73.1–99.1	93.9	281
IBPs	BP2 (OTUs)				66.1–86.6	81.3	315

**Table 5:** Friendly usability of BP1 and BP2. For both BP1 and BP2, a score was assigned according to the sub-criteria reported.

Criteria	Sub-Criteria (SC)	Score 0	Score 1	BP1	BP2
**Computational skills and system requirement (C1)**	Is the pipeline available on Windows? (SC1a)	No	Yes	1	1
	Do you need to have any programming experience to use the pipeline? (SC1b)	Yes	No	0	1
**Data analysis streamlining (C2)**	Can the BP be easily applied to all samples simultaneously? (SC2a)	No	Yes	1	0
	Is it possible to perform output data analysis (i.e., diversity index and plotting of results) on the software hosting the BP? (SC2b)	No	Yes	1	0
**Cost of analysis (C3)**	Is the software used for hosting BPs free of charge? (SC3a)	No	Yes	1	0
	Are there any free tutorials available for using the pipeline? (SC3b)	No	Yes	1	1
**Computational time consumption (C4)**	Which is the faster BP? (SC4a)	Slower	Faster	1	0
**TOT**				6	3

## Data Availability

The original contributions presented in the study are included in the article/Supplementary Material, further inquiries can be directed to the corresponding author.

## Supplementary Materials

The following supporting information can be downloaded at: https://www.mdpi.com/article/10.3390/foods13132102/s1, Table S1: Taxa sequence abundances variation in sample analyzed with BP1 and BP2.
