# Supplementary material for: The “Dry-Lab” Side of Food Authentication: Benchmark of Bioinformatic Pipelines for the Analysis of Metabarcoding Data
Source: Foods. 2024 Jul 1;13(13):2102. doi: 10.3390/foods13132102 (PMC11241536; doi:10.3390/foods13132102)
Supplement: Supplementary file 1 [file foods-13-02102-s001.zip › foods-3022426-supplementary.pdf]

Table S1: Taxa sequence abundances variation in sample analyzed with BP1 and BP2. Only samples in which differences in composition were found are here reported. Each percentage value is related to difference between the sequence abundance obtained after the analysis of the samples with BP1 and BP2

Sample	*A. domesticus*	*A. diaperinus*	*G. locorojo*	*Gryllus. spp.*	*T. molitor*	*G. bimaculatus*	*L. migratoria*	*P. interpunctella*
**IBP-11**	0,27 %	0,27 %	0,00 %	0,00 %	0,00 %	0,00 %	0,00 %	0,00 %
**IBP-12**	0,49 %	0,00 %	0,52 %	0,03 %	0,00 %	0,00 %	0,00 %	0,00 %
**IBP-18**	0,46 %	0,59 %	0,14 %	0,00 %	0,00 %	0,00 %	0,00 %	0,00 %
**IBP-20**	1,54 %	0,00 %	1,54 %	0,00 %	0,00 %	0,00 %	0,00 %	0,00 %
**IBP-21**	0,35 %	0,00 %	0,35 %	0,00 %	0,00 %	0,00 %	0,00 %	0,00 %
**IBP-26**	0,27 %	0,00 %	0,38 %	0,11 %	0,00 %	0,00 %	0,00 %	0,00 %
**IBP-27**	0,39 %	0,00 %	0,68 %	0,00 %	0,00 %	0,00 %	0,00 %	0,00 %
**IBP-28**	0,06%	0,00 %	0,25 %	0,00 %	0,00 %	0,31 %	0,00 %	0,00 %
**IBP-31**	2,00 %	0,00 %	0,00 %	0,00 %	0,00 %	0,00 %	2,00 %	0,00 %
**IBP-4**	0,00 %	3,64 %	0,00 %	0,00 %	2,56 %	0,00 %	1,08 %	0,00 %
**IBP-45**	0,33 %	0,00 %	0,13 %	0,20 %	0,00 %	0,00 %	0,00 %	0,00 %
**IBP-46**	0,40 %	0,00 %	0,01 %	0,38 %	0,00 %	0,00 %	0,00 %	0,00 %
**IBP-6**	0,17 %	0,00 %	0,33 %	0,16 %	0,00 %	0,00 %	0,00 %	0,00 %
**IBP-7**	0,80 %	0,00 %	0,80 %	0,00 %	0,00 %	0,00 %	0,00 %	0,00 %
**IBP-8**	0,00 %	2,18 %	0,00 %	0,00 %	0,00 %	0,00 %	0,00 %	2,18 %
**Average**	0,08 %	1,67 %	0,12 %	0,33 %	2,56 %	0,31 %	1,54 %	2,18 %
